# Stand-Alone and Hybrid Positioning Using Asynchronous Pseudolites

**DOI:** 10.3390/s150100166

**Published:** 2014-12-24

**Authors:** Ciro Gioia, Daniele Borio

**Affiliations:** European Commission, Joint Research Centre (JRC), Institute for the Protection and Security of the Citizen (JRC), Security Technology Assessment Unit, Via Enrico Fermi 2749, 21027 Ispra (VA), Italy; E-Mail: ciro.gioia@jrc.ec.europa.eu

**Keywords:** asynchronous system, GNSS, GPS, loosely-coupled, tightly-coupled

## Abstract

global navigation satellite system (GNSS) receivers are usually unable to achieve satisfactory performance in difficult environments, such as open-pit mines, urban canyons and indoors. Pseudolites have the potential to extend GNSS usage and significantly improve receiver performance in such environments by providing additional navigation signals. This also applies to asynchronous pseudolite systems, where different pseudolites operate in an independent way. Asynchronous pseudolite systems require, however, dedicated strategies in order to properly integrate GNSS and pseudolite measurements. In this paper, several asynchronous pseudolite/GNSS integration strategies are considered: loosely- and tightly-coupled approaches are developed and combined with pseudolite proximity and receiver signal strength (RSS)-based positioning. The performance of the approaches proposed has been tested in different scenarios, including static and kinematic conditions. The tests performed demonstrate that the methods developed are effective techniques for integrating heterogeneous measurements from different sources, such as asynchronous pseudolites and GNSS.

## 1. Introduction

The ever-increasing demand for location-based services (LBSs) in all environments is promoting the development of augmentation systems to aid or replace GNSS. GNSS-based navigation in difficult environments is hindered by signal attenuation and blockage and cannot usually provide the level of performance required. In open-pit mines, urban canyons and indoors, measurements are usually affected by gross errors due to multipath and fading, even when GNSS signals are available. In such conditions, the lack of signals of good quality makes GNSS-based navigation unreliable, if not unfeasible. For this reason, several complementary technologies have been developed to make LBSs available in such environments.

Pseudolites or pseudo-satellites [1,2] are ground-based transmitters able to provide GNSS-like signals in environments where GNSS navigation is precluded due to poor satellite visibility and bad reception conditions. The synergy between GNSS and pseudolites has thus the potential to enable seamless navigation.

Pseudolites have been traditionally used in a synchronous configuration [3,4], where all of the pseudolites are synchronized to a common time scale. In this way, it is possible to use time of arrival (TOA) measurements and trilateration for the computation of the user position. This is the same principle adopted by standard GNSS navigation [5,6]. Synchronization, however, requires a complex architecture, which increases the complexity of the pseudolite system and, consequently, its cost. Thus, alternative solutions have been considered, where each pseudolite operates independently, as described in [7,8]. This has led to the development of asynchronous pseudolite technologies characterized by a simplified system design and reduced costs.

Due to the lack of synchronization between the different pseudolites, the receiver is not able to extract TOA measurements; hence, different techniques have to be adopted to determine the user position. In this paper, two approaches have been considered based on proximity-based location and RSS measurements [9].

When the proximity principle is adopted, the user position is determined as that of the transmitter associated with the strongest received pseudolite signal. This principle has been adopted by the indoor messaging system (IMES) [10] developed by the Japanese Aerospace Exploration Agency (JAXA) as part of the Quasi-Zenith Satellite System (QZSS). When RSS measurements are used, the received pseudolite power is converted into distances using an empirical model [11] whose parameters can be transmitted as part of the pseudolite navigation message.

In [8], meter-level indoor navigation was demonstrated exploiting the potentiality of asynchronous pseudolites. Although both proximity and RSS approaches were considered, the authors did not investigate the potential benefits of integrating GNSS and asynchronous pseudolites measurements. Indoor location was achieved using the information provided only by the pseudolite signals.

In this paper, a hybrid asynchronous pseudolite/GNSS system is developed, and several asynchronous pseudolite/GNSS integration strategies are considered. Hence, a navigation solution integrating both pseudolite and GNSS observations is obtained.

Two different integration strategies are considered:

- loosely-coupled integration, where the integration is performed at the position level. The position computed using the asynchronous pseudolite system is converted into pseudo-measurements [12], which are integrated within the GNSS measurement model
- tightly-coupled integration, where the distances obtained using RSS positioning are used together with GNSS pseudoranges.

Loosely-coupled integration is able to provide a navigation solution also in the case of a complete GNSS outage. In this case, the position solution coincides with that computed using only pseudolite measurements.

Pseudolite-only position can be determined using different approaches. The proximity principle can be generalized by computing the user position as the weighted centroid (WeC) of the different pseudolite locations; such an approach is commonly used in network positioning [13,14] and is adapted here to the pseudolite case. RSS localization is usually adopted for indoor positioning using WiFi signals [15,16]. In this paper, carrier-to-noise density power ratio (*C*/*N*_0_) values, estimated by a GNSS receiver processing pseudolite signals, are used as scaled RSS measurements. The user position is then determined using a modified weighted mean square error (WMSE) criterion. This approach can be considered as an extension of the algorithm discussed in [15,16], which requires an estimate of the measurement variance.

The position solution is then integrated in the GNSS positioning algorithm. In this case, the weighted least squares (WLS) cost function to be minimized to determine the final user position is made of two terms. The first term is due to the pseudoranges obtained by a high sensitivity (HS) GNSS receiver, while the second takes into account the pseudolite solution.

In the tightly-coupled approach, only RSS observations are considered, and the *C*/*N*_0_ measurements of the pseudolite signals are at first converted into distances using an empirical model, as detailed in [8]. The distances are then used together with the pseudoranges obtained from a HS receiver. Furthermore, in this case, the user position is obtained by solving a mixed WLS problem defined by the GNSS pseudorange measurements and the pseudolite distances. In tightly-coupled integration, at least one satellite has to be available in order to estimate the GNSS receiver clock bias.

Several data collections in different environments have been carried out in order to analyze the techniques developed from an experimental point of view.

The algorithms developed were tested in difficult environments, including a large meeting room in an office building made of concrete. Due to the unavailability of a reference trajectory for the indoor tests, the performance of the proposed approaches have been analyzed using qualitative criteria. For example, it has been verified that the position solutions were contained within the test room and respected the constraints imposed by furniture and other obstacles. Moreover, repeatability tests have been conducted in order to assess the consistency among position solutions obtained at different time instants.

Moreover, outdoor tests were conducted: in this case, a reference solution was computed using clear-sky GPS measurements. The performance of the different hybrid navigation solutions have been evaluated in terms of root mean squared (RMS) and maximum errors for both horizontal and vertical components.

The experimental equipment adopted for the tests comprises a commercial off-the-shelf (COTS) pseudolite system from Space System Finland (SSF) [4] and a u-blox LEA 6T HS GNSS receiver.

From the tests, it emerges that the information provided by an asynchronous pseudolite system allows the user to compensate for the limited satellite signal availability and to improve GNSS positioning performance in all of the considered environments.

The methods developed are effective techniques for integrating heterogeneous measurements from different sources, such as asynchronous pseudolites and GNSS. Moreover, the possibility of weighting the measurements according to their quality and type makes the algorithms flexible and suitable for different contexts. The usage of asynchronous pseudolites significantly reduces system costs and alleviates the requirement of having transmitters operating in the same frequency bands as that adopted by GNSS.

This paper is an extended version of [17], where new tests have been performed, and the performance has been assessed using outdoor experiments.

The remainder of this paper is organized as follows: in Section 2, WeC positioning is described and the RSS algorithm is introduced. The integration strategies considered are detailed in Section 3, and the experimental setup adopted for the analysis is detailed in Section 4. The results obtained are described in Section 5, and finally, conclusions are provided in Section 6.

## 2. Asynchronous Pseudolite Positioning

In this section, the techniques adopted for asynchronous pseudolite positioning are briefly reviewed.

### 2.1. Proximity and Weighted Centroid Positioning

The most simple form of positioning based on proximity consists of estimating the receiver position as that of the transmitter associated with the strongest received signals. The WeC approach is an extension of the proximity principle, where the user position is a linear combination of the pseudolite coordinates:

(1) $$P_{u}=\left(x_{u},y_{u},z_{u}\right)=\frac{\sum_{i=1}^{H}w_{i}P_{pl,i}}{\sum_{i=1}^{H}w_{i}}$$

where:

$$P_{pl,i}=\left(x_{i},y_{i},z_{i}\right)$$

is the vector containing the coordinates of the *i*-th pseudolite and *w_i_* is the weight associated with the *i*-th pseudolite. *H* is the number of pseudolites. In this work, the weights are related to the *C*/*N*_0_ of the received pseudolite signal, and the following relationship is adopted:

(2) $$w_{i}=10^{{\left(C/N_{0}\right)}_{i}/10}$$

where (*C*/*N*_0_)*_i_* is the *C*/*N*_0_ of the *i*-th received pseudolite signal expressed in dB-Hz.

### 2.2. RSSI Positioning

RSS is defined as the voltage measured by a receiver signal strength indicator (RSSI) circuit and corresponds to the received power measured in a logarithmic scale [9]. RSS measurements are usually modeled as [9,11,18,19]:

(3) $$P\left(d\right)=P_{\text{ref}}-10\alpha \log_{10}\frac{d}{d_{\text{ref}}}$$

where *P*(*d*) is the received power measured at the distance *d* from the transmitter. *α* is the path-loss exponent and *P_ref_* is the power received at the reference distance, *d_ref_*.

The RSS is easy to measure and can be obtained, for example, from automatic gain control (AGC) levels [18,20,21] or from *C*/*N*_0_ measurements [22].

In this paper, RSSI positioning using *C*/*N*_0_ measurements is considered. In particular, Equation (3) can be rewritten in terms of *C*/*N*_0_ measurements as:

(4) $${\left(\frac{C}{N_{0}}\right)}_{i}=K_{i}-\alpha 10\log_{10}\left(d_{i}\right)$$

where the index, *i*, denotes the *i*-th transmitter and *K_i_* is a constant accounting for the power of the transmitted signals.

When *K_i_* and *α* are known, it is possible to establish a direct relationship between the measured *C*/*N*_0_ and the transmitter-receiver distance. In turn, distances can be expressed as a function of the user position:

(5) $$d_{i}=\sqrt{{\left(x_{u}-x_{i}\right)}^{2}+{\left(y_{u}-y_{i}\right)}^{2}+{\left(z_{u}-z_{i}\right)}^{2}.}$$

Using Equation (5), it is possible to rewrite Equation (4) as:

(6) $${\left(\frac{C}{N_{0}}\right)}_{i}=K_{i}-\frac{1}{2}\alpha 10\log_{10}\left[{\left(x_{u}-x_{i}\right)}^{2}+{\left(y_{u}-y_{i}\right)}^{2}+{\left(z_{u}-z_{i}\right)}^{2}\right]$$

where the user coordinates are the only unknowns. When a sufficiently large number of *C*/*N*_0_ measurements is available (*H* ≥ 3), it is possible to determine the user position solving a minimization problem where the cost function is the mean square error (MSE) between the measured values and the model in the right-hand side of Equation (6) and can be expressed as:

(7) $$J\left(x,y,z\right)=\sum_{i=1}^{H}E_{i}^{2}$$

where:

(8) $$E_{i}={\left(\frac{C}{N_{0}}\right)}_{i}-K_{i}+\frac{1}{2}\alpha 10\log_{10}\left[{\left(x-x_{i}\right)}^{2}+{\left(y-y_{i}\right)}^{2}+{\left(z_{u}-z_{i}\right)}^{2}\right].$$

In this way, the user coordinates are computed as:

(9) $$\left(x_{u},y_{u},z_{u}\right)=\arg\underset{x,y,z}{\min}J\left(x,y,z\right).$$

The minimization problem in Equation (9) is solved using a gradient descent algorithm where the initial user position is set equal to the average of the pseudolite coordinates.

The MSE algorithm detailed above was tested using the *C*/*N*_0_ measurements collected in different environments from deep indoors to outdoors. In all of the tests carried out, significant errors were observed when *C*/*N*_0_ values approach zero. This effect reflects the fact that low *C*/*N*_0_ measurements are unreliable. Hence, measurements with *C*/*N*_0_ values close to zero should be removed. For this reason, cost function in Equation (7) was modified to de-weight measurements characterized by low *C*/*N*_0_ values. The new cost function is:

(10) $$J_{w}\left(x,y,z\right)=\sum_{i=1}^{H}{\left(\frac{C}{N_{0}}\right)}_{i}E_{i}^{2}$$

where the subscript “w” denotes the fact that the cost function is a form of WMSE, where each term in the summation in Equation (10) is weighted by its relative *C*/*N*_0_.

The approach detailed above assumes the knowledge of the parameters:

(11) $$\alpha ,K_{i}\;\text{for}\;i=0,\ldots ,H-1.$$

These parameters are, however, unknown and have to be determined using a calibration process. Calibration was performed using *C*/*N*_0_ measurements collected in known positions. Additional details on the calibration procedure adopted for determining parameters can be found in [12].

The approaches mentioned above use *C*/*N*_0_ measurements as input. Such measurements are, however, noisy, and significant performance improvement can be obtained by pre-filtering them. The benefits of *C*/*N*_0_ filtering has been investigated in [7], and a comparison between filtered and unfiltered solutions is provided in Section 5.3. If not differently specified, the *C*/*N*_0_ measurements are at first pre-processed using a filter with a triangular impulse response of a length of five. Justifications for the usage of such a filter can be found in [7].

Using this approach, it was finally possible to perform indoor location using *C*/*N*_0_ measurements.

## 3. Hybrid Positioning Strategy

In this section, different integration strategies are analyzed. The methodologies discussed are: loosely-coupled integration (Section 3.1) and tightly-coupled integration (Section 3.2).

### 3.1. Loosely-Coupled Integration

In the loosely-coupled approach, integration is performed at the position level. The method discussed in this work is the conditional least squares (LS) adjustment with extra conditions; these conditions are included, as additional equations, in the measurement model [6] and constrain the system dynamics. With this approach, the conditions can be considered as measurements or pseudo-measurements.

The conditions are obtained from the asynchronous pseudolite navigation solution and are known with certain *a priori* accuracies, which are used as weights in a WLS algorithm.

Usually the constraints are obtained by considerations on the behavior of the unknowns, for example the coordinates of the user position or the receiver clock bias. Examples of such constraints can be found in [23], where the authors adopted pseudo-measurements modeling the inter-system bias between GPS and GLONASS. Moreover, when the user altitude varies slowly, a pseudo-measurement imposing small altitude variations can be used [24,25].

In this case, the following approach has been adopted. The user position has been at first estimated using the techniques detailed in Section 2.2. Position *P_u_* = (*e_u_*, *n_u_*, *u_u_*) is converted in terms of latitude, longitude and height:

$$P_{u}=\left(\varphi_{u},\lambda_{u},h_{u}\right)$$

and used to introduce constraints on the pure GNSS position solution. The conversion is adopted since the position provided by a GNSS receiver is usually provided in terms of latitude, longitude and height. The user position is computed in an iterative way, linearizing the LS problem. In particular, an initial position, *P_0_* = (*φ*_0_, *λ*_0_, *h*_0_), is selected and updated at each iteration. In the following, *P_n_* denotes the final user position at the *n-th* iteration. This position is distinct from *P_u_*, the user position obtained from the pseudolite system alone.

The position errors with respect to the pure pseudolite solution are given by:

(12) $$\Delta m_{n}=\left[\begin{array}{c} \varphi_{u}-\varphi_{n-1}\\ \lambda_{u}-\lambda_{n-1}\\ h_{u}-h_{n-1} \end{array}\right]$$

where the subscript (*n* − 1) denotes quantities obtained at the (*n* − 1)-th iteration step. Measurements in Equation (12) are related to the state correction vector by:

(13) $$\Delta m_{n}=\left[\begin{array}{cccc} 0 & \frac{1}{M+h_{n-1}} & 0 & 0\\ \frac{1}{\left(N+h_{n-1})\cdot \cos(\varphi_{n-1}\right)} & 0 & 0 & 0\\ 0 & 0 & 1 & 0 \end{array}\right]\;\left[\begin{array}{c} \Delta e_{n}\\ \Delta n_{n}\\ \Delta u_{n}\\ \Delta {\left(\text{cdt}\right)}_{n} \end{array}\right]$$

where:

- *M* is the meridian radius.
- *N* is the radius of curvature in the prime vertical.
- Δ*e_n_*, Δ*n_n_*, Δ*u_n_* and Δ(*cdt*)*_n_* are the errors to add to the nominal states in order to obtain the updated solution.
- *cdt* is the receiver clock bias.

In a standard GNSS algorithm, pseudorange errors are computed with respect to pseudoranges predicted using the (*n* − 1)-th position solution. Similarly to Equation (13), pseudorange differences are related to the state correction vector by:

(14) $$\delta \rho_{n}=\left[\begin{array}{c} \delta \rho_{1,n}\\ \delta \rho_{2,n}\\ \vdots \\ \delta \rho_{K,n} \end{array}\right]=H_{\text{GNSS}}\left[\begin{array}{c} \Delta e_{n}\\ \Delta n_{n}\\ \Delta u_{n}\\ \Delta {\left(\text{cdt}\right)}_{n} \end{array}\right]$$

where *H_GNSS_* is the standard design matrix obtained from the satellite ephemerides [5] and *K* is the number of pseudoranges available. Equations (13) and (14) form a single system of equations, which can be inverted to compute corrections to the state vector. Those corrections are then used to update the user position, *P_n_*. This process is iterated till convergence.

**Figure 1.**
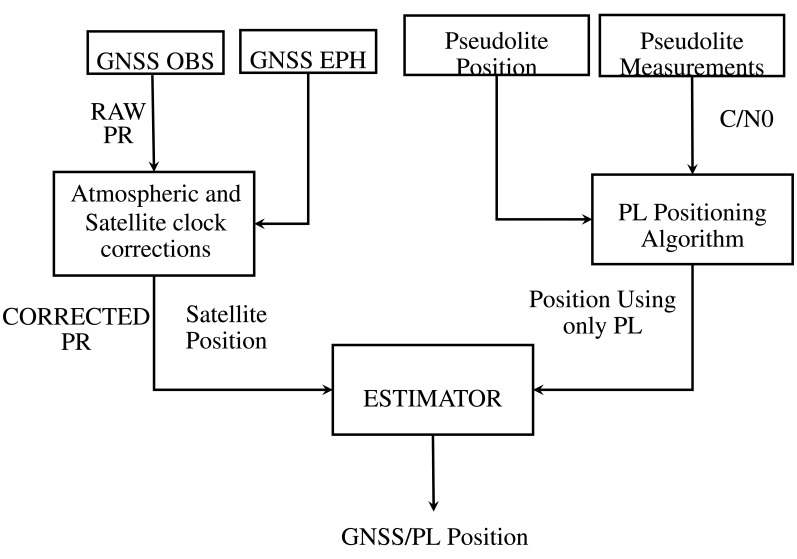
Loosely-coupled integration flow chart.

The impact of the constraints in Equation (13) are weighted relatively to the level of confidence provided to the pseudolite solution. This approach can be considered as a form of loosely-coupled integration, since the position derived from the asynchronous pseudolite system is used to improve the GNSS solution. The flow chart of the algorithm proposed using a loosely-coupled integration strategy is shown in Figure 1.

### 3.2. Tightly-Coupled Integration

**Figure 2.**
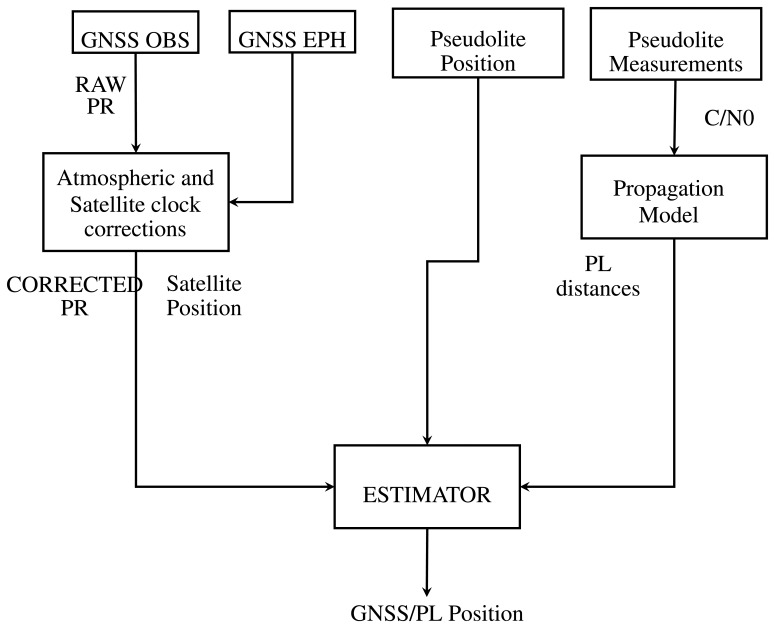
Tightly-coupled integration flow chart.

In order to fully exploit asynchronous pseudolites, a second integration strategy is proposed. This method exploits the measurements obtained from the RSS positioning algorithms described in Section 2.2, which allows the estimation of the distances from each pseudolite. Hence, pseudolite distances can be used directly in the GNSS measurement model. The flow chart of the algorithm proposed for tightly-coupled integration is shown in Figure 2. In this case, the vector containing the differences between the actual and predicted measurements is given by:

(15) $$\delta {\bar{\rho }}_{n}=\left[\begin{array}{c} \delta \rho_{1,n}\\ \delta \rho_{2,n}\\ \vdots \\ \delta \rho_{K,n}\\ \delta d_{1,n}\\ \vdots \\ \delta d_{H,n} \end{array}\right]$$

where Δ*d_i_* are the errors computed with respect to the pseudolite distances.

δ*ρ̄_n_* is used to compute the navigation solution with an iterative WLS. The design matrix of the hybrid system is made of two blocks: the first is related to GNSS pseudoranges and coincides with *H_GNSS_* introduced above. The second block is related to the pseudolite distances. The design matrix of the hybrid system is thus expressed by the following formula:

(16) $$H_{\text{Hyb}}=\left[\begin{array}{cccc} a_{\text{GNS}S_{1}} & b_{\text{GNS}S_{1}} & c_{\text{GNS}S_{1}} & 1\\ a_{\text{GNS}S_{2}} & b_{\text{GNS}S_{2}} & c_{\text{GNS}S_{2}} & 1\\ \vdots & \vdots & \vdots & \vdots \\ a_{\text{GNS}S_{j}} & b_{\text{GNS}S_{j}} & c_{\text{GNS}S_{j}} & 1\\ a_{PL_{1}} & b_{PL_{1}} & c_{PL_{1}} & 0\\ \vdots & \vdots & \vdots & \vdots \\ a_{PL_{H}} & b_{PL_{H}} & c_{PL_{H}} & 0 \end{array}\right]$$

where *a*, *b*, *c* are the cosine directors of the vector from the receiver position to the device that broadcast the signal, either satellite or pseudolite. The solution is obtained using a WLS estimation technique where the weights of the pseudolite distances were empirically determined.

Using the above-mentioned strategy, the integration is performed at the measurement level; hence, it can be considered as a form of tightly-coupled integration.

## 4. Experimental Setup

In this section, the experimental setup adopted for the different tests is described. Several data collections were conducted in different scenarios, from outdoors to deep indoors, in static and kinematic conditions.

**Figure 3.**
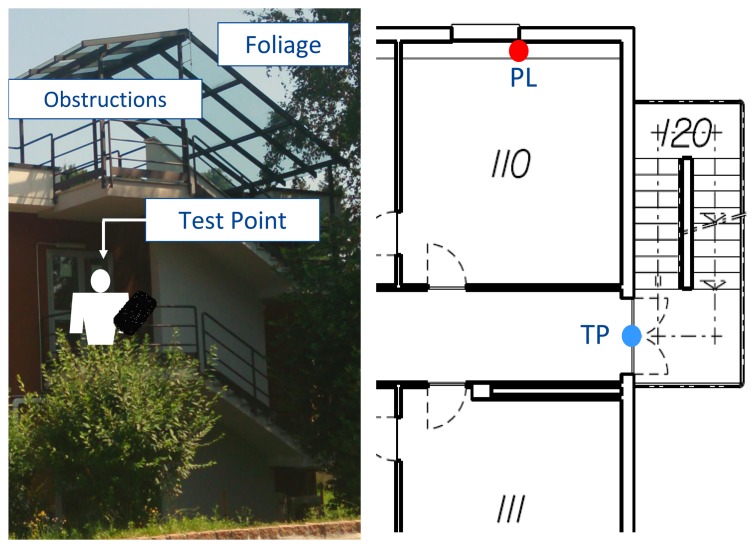
Partially-obstructed scenario selected for testing the improvements brought by a single pseudolite. Marker “TP” indicates the position of the static receiver.

The first dataset was collected in static conditions: the receiver was placed on a balcony at the entrance of the first floor of an office building. Such an environment was classified as a partially-obstructed scenario, because GNSS signals were partially blocked by the building and by the nearby trees. A view of the environment selected is provided in Figure 3 along with the layout of the first floor of the building where the experiment took place. The pseudolite was placed inside an office in the proximity of the entrance of the building. Additional details on the environment selected can be found in [8]. As it emerges from Figure 3, the presence of a roof above the balcony and of several trees on the side of the building introduces gross errors in the measurements, which were affected by multipath and fading. Only one pseudolite was used, and only loosely coupling using proximity information has been considered.

**Figure 4.**
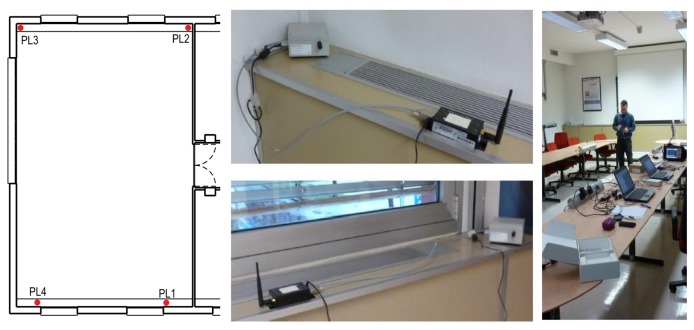
Deep indoor scenario: tests have been conducted in a large meeting room (about 10 × 7 m).

The pseudolite adopted was the one described in [8] and implemented on a Universal Software Radio Platform (USRP); as the measurement unit, a LEA-6T u-blox receiver with a patch antenna was used; the data were collected using an Android mobile phone. The LEA-6T u-blox receiver and the patch antenna were used for all of the tests carried out. The performance of the hybrid system is evaluated in terms of RMS and maximum errors for both horizontal and vertical components. In order to have a reference position to assess the performance of the system under test, a pre-survey was performed using a geodetic technique to determine the coordinates of the reference point where the receiver antenna was placed. The second dataset belongs to a set of data collections performed in the large meeting room (about 10 × 7 m) shown in Figure 4.

This scenario has been classified as deep indoors, since GNSS signals were strongly attenuated by walls. For this environment a reference trajectory was not available; hence, repeatability tests were performed. During these tests, the user carried out several loops around a large table placed in the middle of the meeting room, repeating the same trajectory. The quality of the navigation solution was assessed by comparing the different trajectories estimated for the different loops. A high consistency level of the navigation solution indicates the good performance of the system. In this case, the pseudolite system adopted is COTS produced by SSF [4]. The system is composed by:

- 4 pseudolites able to broadcast continuous and pulsed signals placed in the corners of the room;
- several radio modems, which allow one to configure and control the pseudolites.

**Figure 5.**
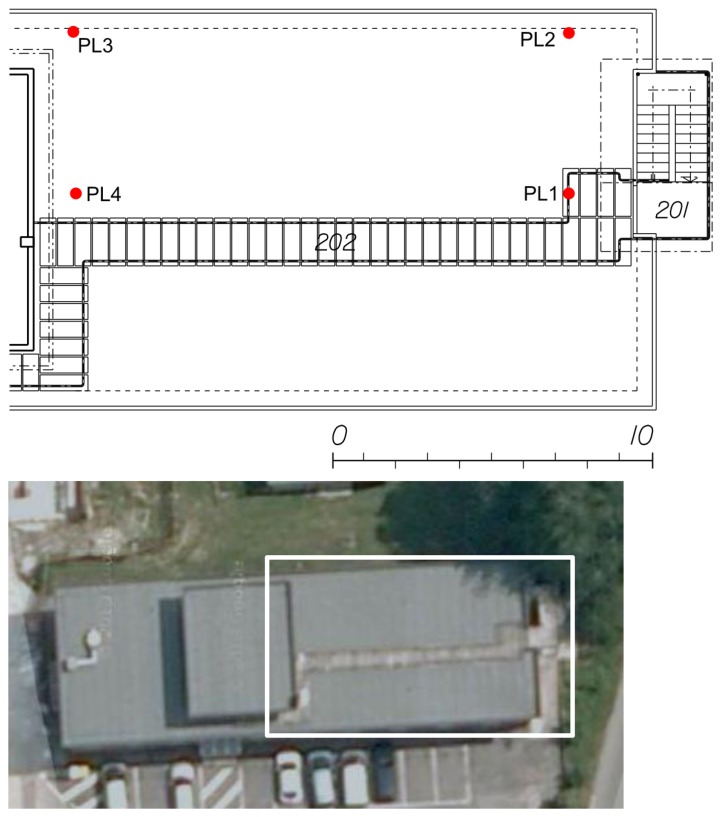
Outdoor scenario selected for the performance evaluation of hybrid pseudolite/GNSS positioning.

A view of the SSF pseudolites is provided in Figure 4 along with the location of the transmitters. In order to have a performance evaluation of the proposed architectures in kinematic conditions, a third data collection was carried out on the rooftop of the office building already considered for the other tests. The SSF pseudolite system was deployed on the rooftop of the building according to the geometry described in Figure 5: four pseudolites were placed in the corners of a rectangular area of about 15 × 7 m. The tests were carried out inside the rectangle defined by the pseudolites.

## 5. Experimental Results

In the following sections, results obtained using pseudolite/GNSS hybrid systems are presented. First of all, the results obtained in the partially-obstructed scenario and static conditions are described; then, the results obtained in deep indoor environments are presented, and finally, an assessment of the system performance outdoors is performed.

### 5.1. Partially-Obstructed Scenario

**Figure 6.**
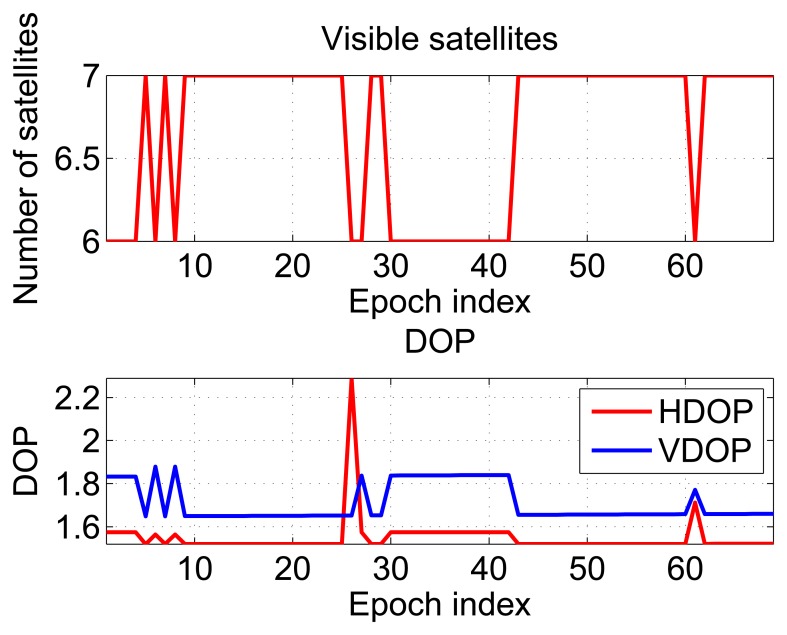
Number of visible satellites and dilutions of precision (DOPs) recorded during the test conducted in the partially-obstructed scenario.

In the partially-obstructed scenario, only the loose integration strategy using proximity information is discussed. This is due to the availability of a single pseudolite. The performance of the hybrid system is compared to the GPS-only solution. Horizontal and vertical errors of both configurations are evaluated. During the test, the user was static for almost 100 seconds, and the number of visible satellites varied between six and seven, as shown in the upper plot of Figure 6.

**Figure 7.**
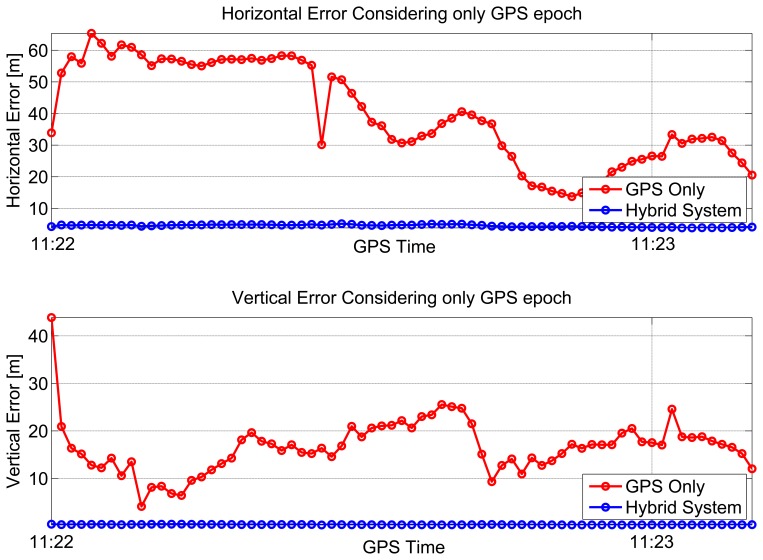
Horizontal and vertical errors for the partially-obstructed scenario.

**Figure 8.**
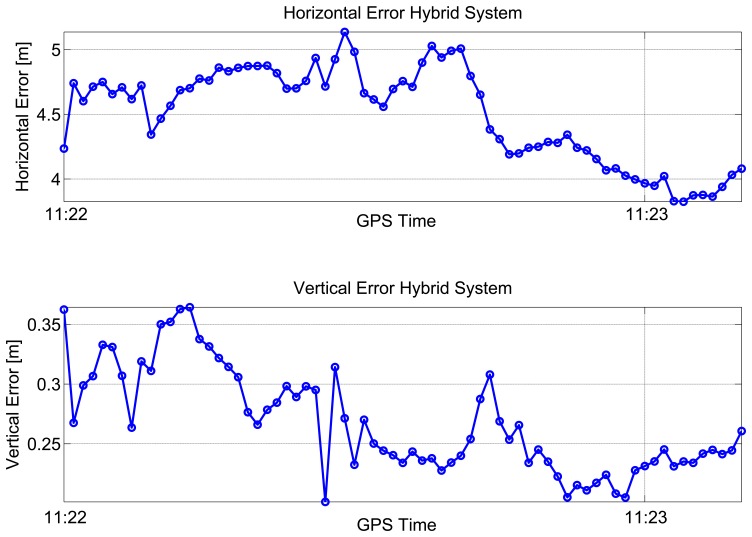
Horizontal and vertical errors of the hybrid solution with proximity information.

In the bottom part of Figure 6, the dilution of precisions (DOPs) are plotted as a function of time: the mean values of the horizontal dilution of precision (HDOP) and vertical dilution of precision (VDOP) were 1.77 and 1.87, respectively. Horizontal (upper box) and vertical (lower box) position errors of the considered configurations are plotted as a function of time in Figure 7. From the upper box, the benefit of including pseudolite information clearly emerges. Even if a single pseudolite is used, the hybrid system (blue line) provides better performance with respect to GPS alone (red line). The horizontal error reaches a maximum value of 65 m in the case of GPS-only. Such a value is reduced to 5.1 m when pseudolite measurements are introduced. Despite the good geometry conditions shown in Figure 6, the poor quality of the measurements makes GPS-only positioning unreliable. The benefits of the hybrid system clearly emerge also analyzing the RMS value, which is reduced almost ten times in the case of the hybrid solution passing from 43 to 4.5 m, as detailed in Table 1. The hybrid system provides even better results in the vertical component: the vertical error is bounded to 0.4 m in the case of the hybrid system, while the maximum error for the GPS-only configuration reaches 43 m. Horizontal and vertical errors of the hybrid system are plotted as a function of time in Figure 8: from the figure, it clearly emerges that the error is not only related to the distance between the receiver and the pseudolite, but also to the relative weight between pseudolite and GNSS measurements. The weights were empirically selected as a compromise between bounding the maximum error and system reactivity to position changes. This explains why the maximum error is slightly greater than the distance between the user and the pseudolite position. Statistical parameters of both horizontal and vertical errors for the considered configurations are summarized in Table 1.

### 5.2. Deep Indoors

**Figure 9.**
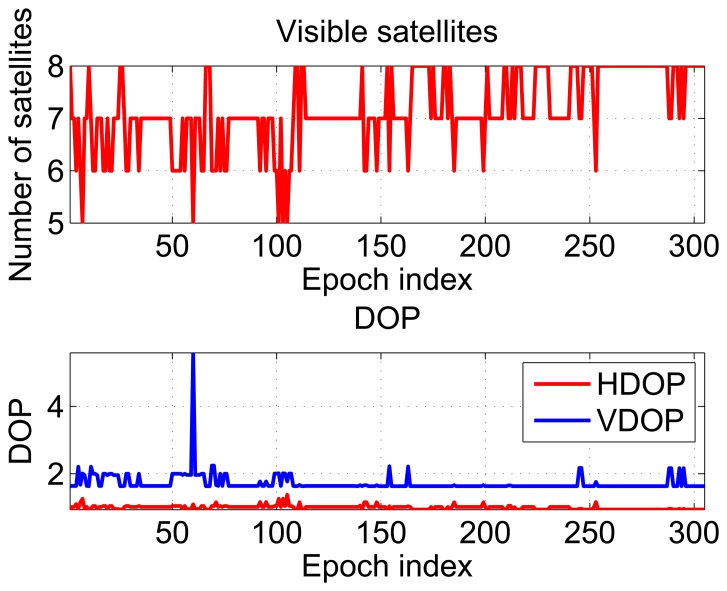
Number of visible satellites and DOPs recorded during the test conducted in the meeting room.

In order to have an evaluation of the performance of the hybrid system in kinematic conditions, the results obtained in the indoor scenario described in Section 4 are detailed. The receiver used was able to track up to eight satellites. The receiver is HS and has the capability of tracking very weak satellite signals. This fact clearly emerges from Figure 9, which shows the number of satellites tracked and the DOPs as a function of time.

**Figure 10.**
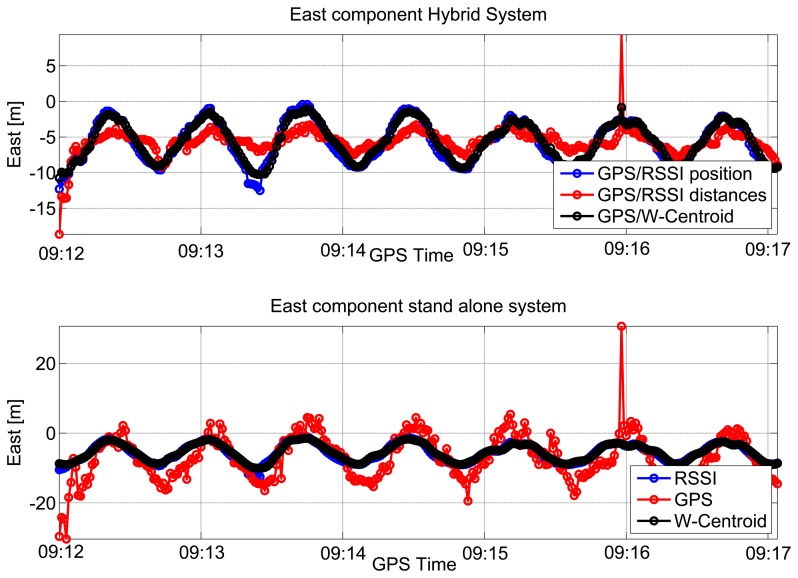
East component estimated for the repeatability test performed in a large meeting room.

**Figure 11.**
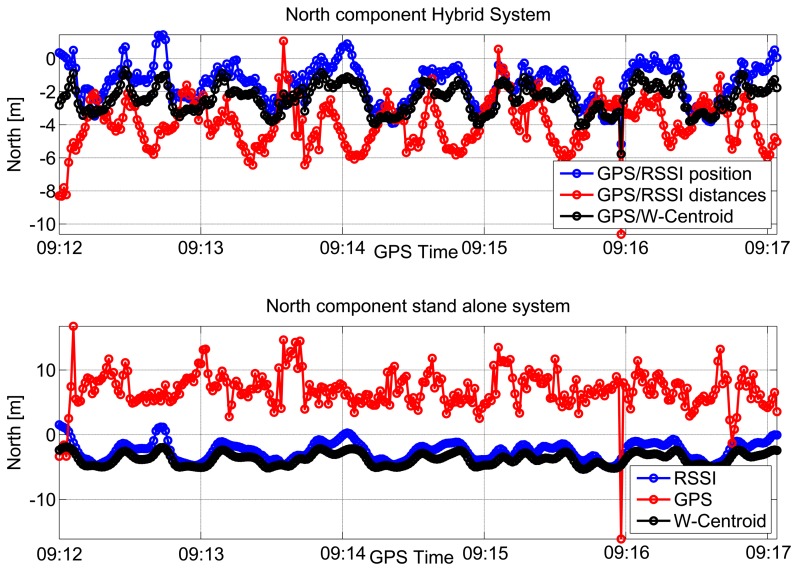
North component estimated for the repeatability test performed in a large meeting room.

As for the partially-obstructed scenario, these metrics do not take into account the poor quality of the measurements and provide an overoptimistic description of the testing environment. The results of the repeatability tests are shown in Figures 10 and 11, respectively. In the upper box of Figure 10, east coordinates estimated using the hybrid systems are plotted as a function of time; three different configurations are considered to differ for the integration strategy, *i.e.*, loose or tight, and for the algorithm adopted for the pseudolite-only solution, *i.e.*, WeC or RSS. From the figure, a periodic pattern can be clearly identified showing the different laps performed by the user; sub-meter differences can be appreciated between the three configurations. From the lower box of Figure 10, it emerges that also the GPS-only solution is characterized by a periodic behavior. This result is probably due to the presence of windows on the two opposite sides of the room along the east-west direction. In this way, the receiver had sufficiently good geometry conditions, and it was able to discriminate the user motion along the east direction.

The north coordinates computed using the hybrid system are shown in the upper box of Figure 11 as a function of time. Furthermore, in this case, three different approaches have been considered. As for the east component, a periodic pattern can be observed when considering hybrid navigation systems. This periodic behavior is less evident than in the east case. The north coordinates estimated using standalone systems are plotted as a function of time in the lower box of Figure 11. From the figure, it clearly emerges that only the standalone GPS solution (red line) is significantly degraded and biased. This is due to the geometry of the room, which does not have windows on the north and south sides.

**Figure 12.**
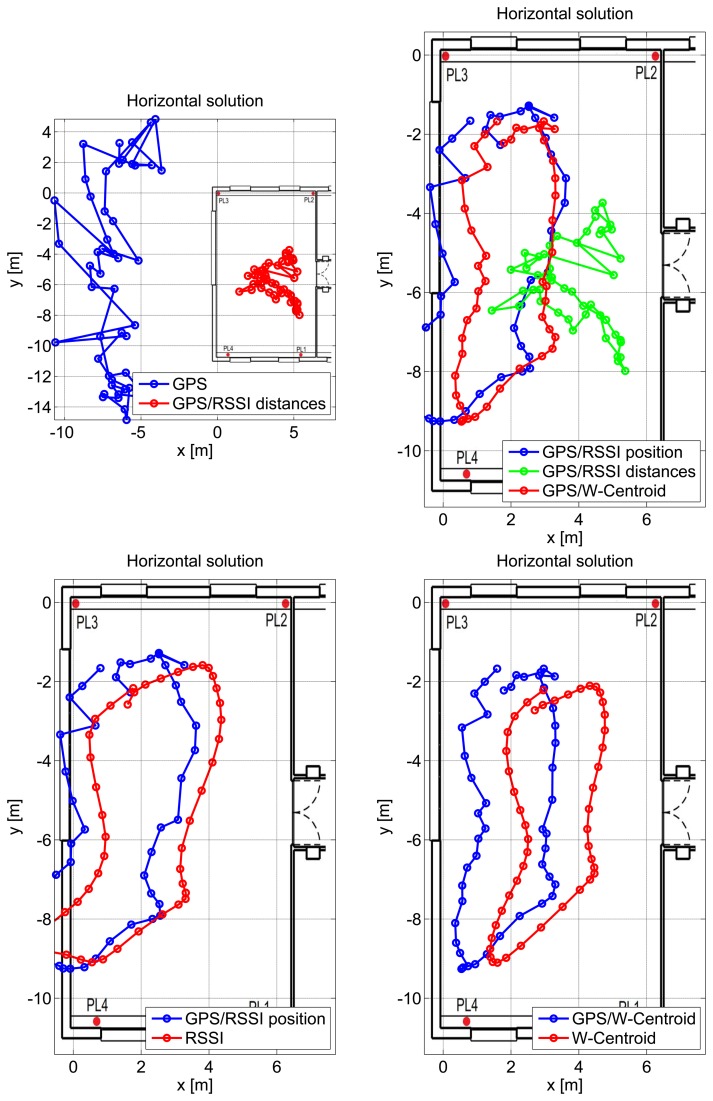
Horizontal solution estimated for the repeatability test performed in a large meeting room. A single lap is provided for improving the clarity of the representation.

From the results provided in Figure 12, it clearly emerges that GPS measurements are significantly biased in indoor environments, and thus, a pseudolite-only navigation solution should be preferred. In the case considered here, the WeC solution provides the best performance.

In the RSSI-only case considered in the bottom left plot of Figure 12, a small bias can be observed. The user is, however, located inside the room for most of the time, and the loop performed can be clearly identified. The three hybrid solutions are compared in the top right plot of Figure 12. As already mentioned, the best performance is obtained when considering integration with the WeC solution.

The different solutions are compared in the horizontal plane in Figure 12. In this case, a single lap is provided for improving the clarity of the representation. The GPS-only solution is significantly biased along the north/south direction, and the user trajectory is estimated outside the room. The introduction of RSS measurements in a tightly-coupled solution significantly reduces the GPS error, and the user is correctly located inside the room. Despite the improvement provided by RSS measurements, it is not possible to distinguish the loop performed by the user. Loosely-coupled integration is considered in the bottom plots of Figure 12 along with the pseudolite-only solutions. From a qualitative point of view, the WeC approach provides the best position performance: the user is always located inside the room, and the trajectory changes imposed by the presence of furniture and other obstacles are clearly respected. The inclusion of GPS measurements in the combined solution introduces a small bias towards the north direction. This phenomenon is expected, since GPS measurements are significantly biased towards north.

### 5.3. Rooftop Experiment

**Figure 13.**
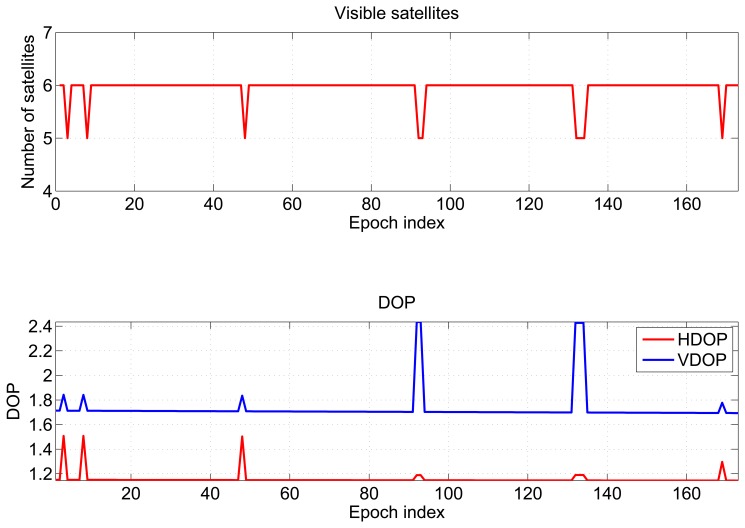
Number of visible satellites and DOPs recorded during the test conducted in the outdoor scenario.

Results obtained in the repeatability tests performed outdoors are presented in this section. The number of visible satellites varies between five and six, as shown in the upper box of Figure 13. The HDOP and VDOP are plotted as a function of time in the lower box of Figure 13; the mean values are 1.15 and 1.72, respectively.

Although the number of satellites tracked is lower than in the indoor case, the receiver has a more constant behavior, and almost no loss of lock occurs.

At first, the performance of standalone pseudolite positioning is evaluated; then, the benefits of asynchronous pseudolite integration are investigated.

**Figure 14.**
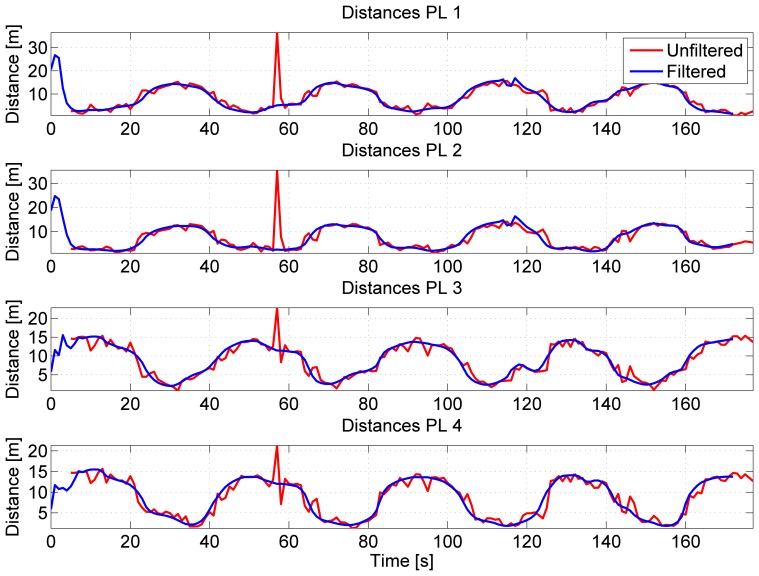
Distances estimated from the *C*/*N*_0_ measurements of the four outdoor pseudolites. Filtering clearly improves the performance of the system.

During the test, the user performed four laps on the rooftop of an office building: the periodic behavior of the activity performed by the user can be clearly seen in the distances estimated from the four pseudolites using the RSS algorithm described in Section 2.2. The distances from the four pseudolites are plotted as a function of time in Figure 14. From the figure, the effect of *C*/*N*_0_ filtering clearly emerges: after the first lap, an erroneous measurement is observed in the unfiltered distances (red lines). Such error is mitigated by *C*/*N*_0_ filtering, and it is not evident in the filtered distances (blue lines). This result confirms the findings obtained in [7].

**Figure 15.**
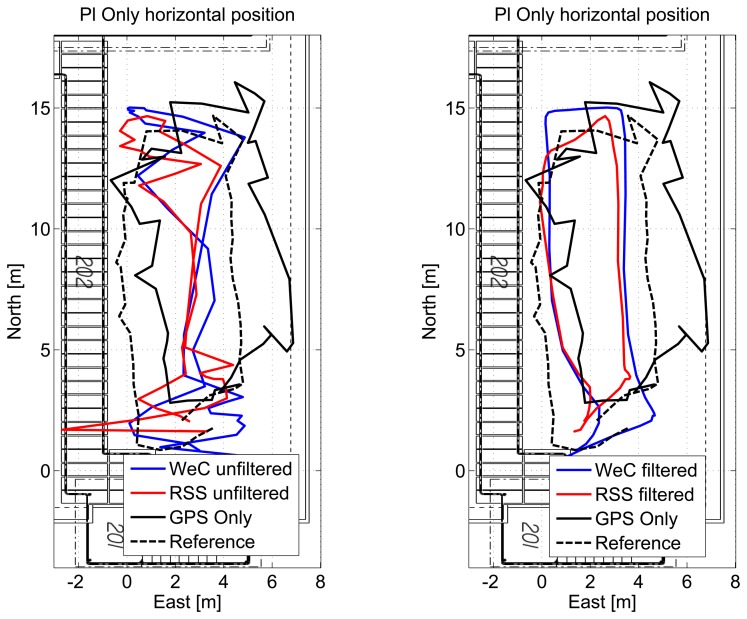
Pseudolite and GPS-only solutions obtained outdoors. A single lap is provided for clarity.

The horizontal solutions using only pseudolite measurements are shown in Figure 15 for a single lap. The solution obtained using unfiltered measurement is shown in the left box: from the figure, it emerges that although the unfiltered solution is within the area of the test, it is not possible to identify user trajectory. In the right box, where filtering is applied, the user trajectory can be clearly identified. In order to have a complete analysis of the system performance, the unfiltered and filtered solutions are compared to the GPS-only solution (black dashed line) and to the reference trajectory (black line).

**Figure 16.**
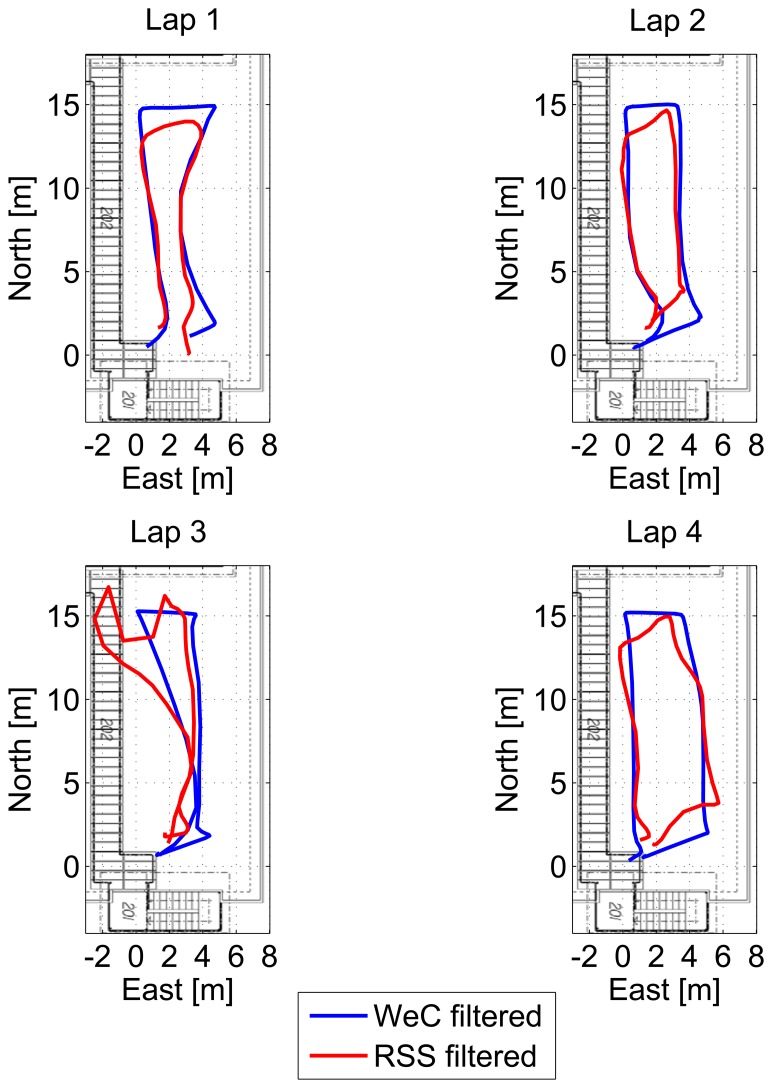
Trajectories obtained considering the weighted centroid (WeC) and RSS approaches for the four laps performed by the user.

The trajectories obtained considering the four laps are provided in Figure 16 for the WeC and RSS approaches: only small differences can be noted between the different laps. Even during the third lap, where the trajectory is slightly distorted, an accuracy of metric order is obtained. Statistical error parameters of the pseudolite-only solutions are summarized in Table 2. From the table, it clearly emerges that the filtering effect not only limits the maximum error, but also reduces the RMS error for the considered configurations. The RSS and WeC algorithms have a similar performance in terms of horizontal RMS error, and only a difference of 25 cm is measured. However, the WeC approach provides a reduction of two meters in the maximum error.

**Figure 17.**
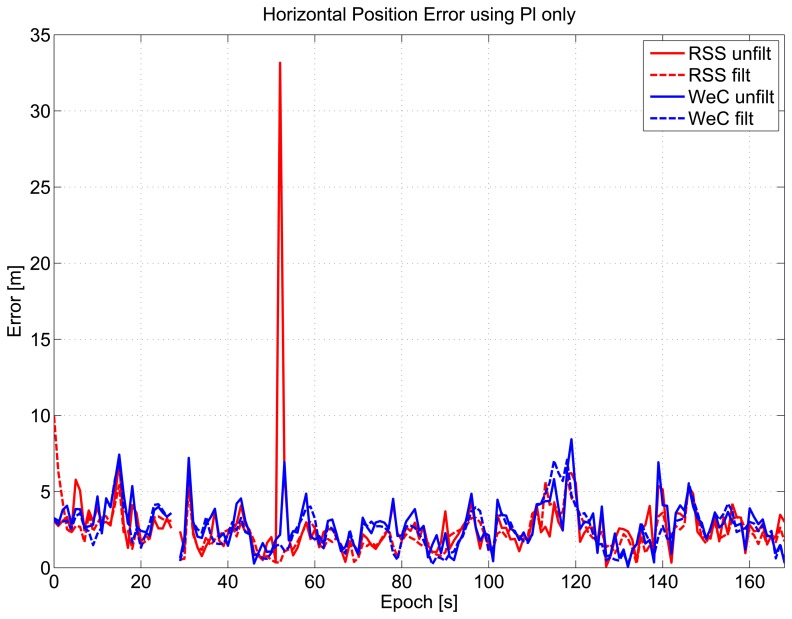
Horizontal error as a function of time, pseudolite-only solutions.

Horizontal position errors of the four pseudolite-only configurations are plotted as a function of time in Figure 17: the filtered configurations (red and blue dashed lines) have errors with lower values than the corresponding unfiltered configurations. The effect of measurement errors, highlighted in Figure 14, is less evident.

**Figure 18.**
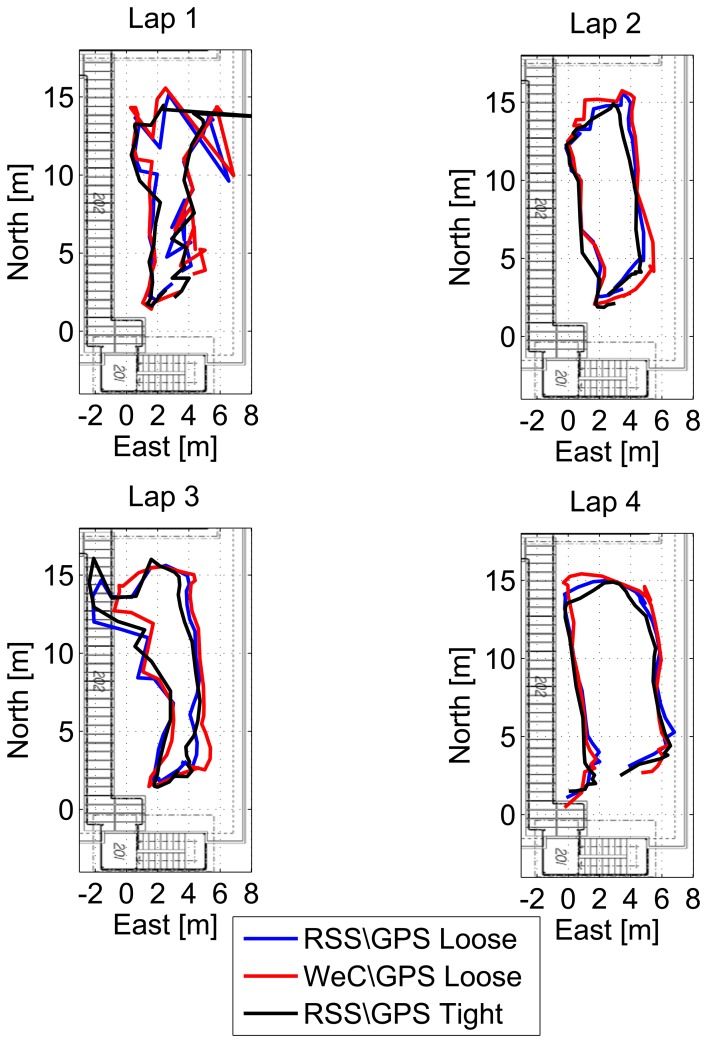
Trajectories obtained considering hybrid approaches for the four laps performed by the user.

The horizontal solutions for each single lap are shown in Figure 18 for the different configurations of the hybrid system. The trajectory is properly reconstructed in all of the laps, and only small differences between the architectures proposed can be appreciated. Even in the case of the hybrid system, the third lap shows some inaccurate positioning; however, the user position is inside the area of the test, and the trajectory can be identified.

**Figure 19.**
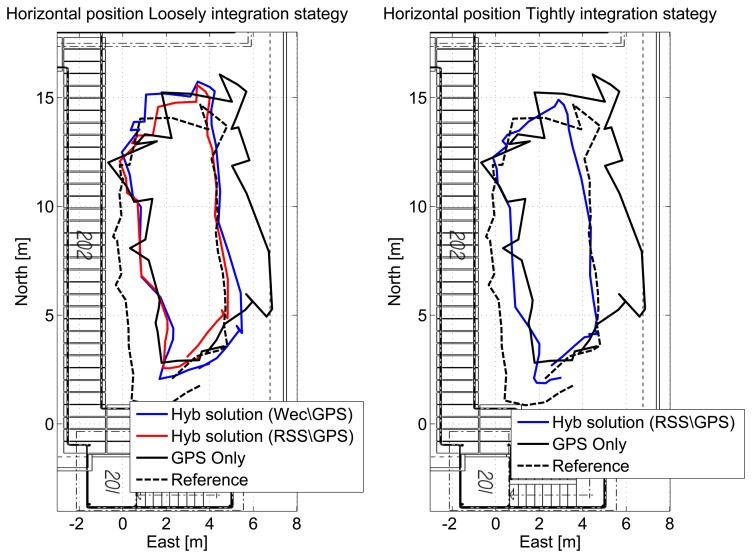
Trajectories obtained using hybrid integration strategies in the outdoor scenario. A single lap is provided for clarity.

In order to have a better representation of the performance achievable by the hybrid system, the horizontal solutions for a single lap are shown in Figure 19 for the different configurations. In the left box of Figure 19, the solutions obtained using loosely-coupled integration strategies (red and blue lines) are compared to the GPS-only (black line) position fixes and to the reference solution (black dashed line) obtained using a real-time kinematic (RTK) technique. In the right box of Figure 19, the solution obtained using the tightly-coupled integration strategy is compared to the GPS-only case and to the reference trajectory. From the figures, the benefits of the hybrid system clearly emerge: the trajectory of the hybrid solutions are closer to the reference. In the same points, the trajectory obtained with the hybrid system seems to be even more reliable than the reference one, since jumps are not present.

**Figure 20.**
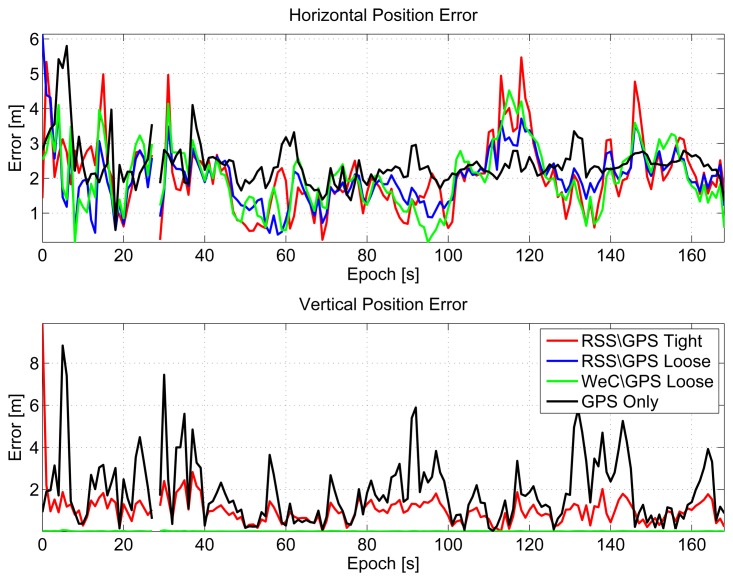
Horizontal and vertical errors as a function of time, hybrid solutions.

In Figure 20, horizontal (upper box) and vertical (lower box) errors of the considered configurations are plotted as a function of time. From the upper box, the benefits of the inclusion of pseudolite information clearly emerge. Error lines representing the hybrid solutions are lower than the black line, the GPS-only solution. The improvement is more clear in the vertical component, where the reference solution is considered constant and equal to the altitude of the pseudolites.

Statistical error parameters for both horizontal and vertical components are summarized in Table 3. The inclusion of pseudolite measurements reduces the RMS horizontal error, by almost 20 cm. The improvement in the vertical channel is more evident, and the RMS error is reduced by almost one meter.

## 6. Conclusions

In this paper, the performance of an asynchronous pseudolite system was investigated in static and kinematic conditions and in different scenarios, including outdoors and deep indoors. Two asynchronous localization approaches were adapted to pseudolite positioning and used for indoor navigation. The algorithms are based on WeC and RSS, respectively: in both cases, the user position is computed exploiting *C*/*N*_0_ measurements from different pseudolites.

Indoor navigation, with meter-level accuracy, has been demonstrated using the asynchronous pseudolite system, and the benefits of *C*/*N*_0_ pre-filtering have been evaluated for an outdoor scenario.

In order to fully exploit the potential of the asynchronous pseudolite system, hybrid pseudolite/GNSS integration strategies were suggested, and the performance was evaluated in the scenarios mentioned above. Three different architectures were considered, including two forms of loosely-coupled integration. The third approach was a tightly-coupled integration strategy.

From the analysis, it emerges that the integration with pseudolite measurements can significantly reduce the horizontal and vertical error with respect to the GPS-only solution. The actual improvement depends on the scenario considered. For example, in a partially-obstructed scenario, the maximum positioning error is reduced by a factor of ten when integration is performed.

From indoor tests, it emerges that GNSS navigation is not feasible indoors and that GPS-only solutions are significantly biased. Although, in a hybrid configuration, the pseudolite/GNSS system is able to provide meter level accuracy, pseudolite-only navigation should be preferred. The GPS measurements are significantly biased and should not be trusted when computing the hybrid solution. In the large meeting room considered, the best performance was achieved using the WeC solution without GPS hybridization.

In the outdoor tests, only sub-meter differences were observed among the configurations considered; hence, the strategy adopted for integration seems to have a limited impact. However, loose coupling provides a more continuous solution with respect to the tight strategy. The first strategy is able to provide navigation solution, also in the case of a complete GNSS outage. Under such conditions, the position estimated coincides with the pseudolite-only solution. In tightly-coupled integration, at least one satellite has to be available in order to estimate the GNSS receiver clock bias.

The results obtained using pseudolite/GNSS hybrid systems demonstrate that the synergy between GNSS and pseudolites has the potential to enable seamless navigation.

## Figures and Tables

**Table 1.** Hybrid pseudolite/GPS statistical position error parameters: RMS and maximum errors for both horizontal and vertical components for the partially obstructed scenario.

**Configuration**	**RMS (m)**		**Max (m)**	
	Horizontal	Vertical	Horizontal	Vertical
GPS Only	42.78	17.54	65.36	43.86
Pseudolite/GPS using proximity information	4.52	0.27	5.14	0.36

**Table 2.** Pseudolite-only statistical position error parameters: RMS and maximum errors for the horizontal component.

**Configuration**	**RMS (m)**	**Max (m)**
	Horizontal	Horizontal
RSS using raw measurements	3.82	33.16
RSS using filtered measurements	2.66	9.96
WeC using raw measurements	3.16	8.44
WeC using filtered measurements	2.91	7.09

**Table 3.** Hybrid pseudolite/GPS statistical position error parameters: RMS and maximum errors for both horizontal and vertical components.

**Configuration**	**RMS (m)**		**Max (m)**	
	Horizontal	Vertical	Horizontal	Vertical
GPS Only	2.48	2.45	5.80	8.83
RSS/GPS Loosely-Coupled Integration	2.17	0.01	6.14	0.07
WeC/GPS Loosely-Coupled Integration	2.21	0.01	4.51	0.07
RSS/GPS Tightly-Coupled Integration	2.28	1.34	5.47	9.88
